# Supplementary material for: Co-Zeolitic Imidazolate Framework@Cellulose Aerogels from Sugarcane Bagasse for Activating Peroxymonosulfate to Degrade P-Nitrophenol
Source: Polymers (Basel). 2021 Feb 27;13(5):739. doi: 10.3390/polym13050739 (PMC7957538; doi:10.3390/polym13050739)
Supplement: Supplementary file 1 [file polymers-13-00739-s001.pdf]

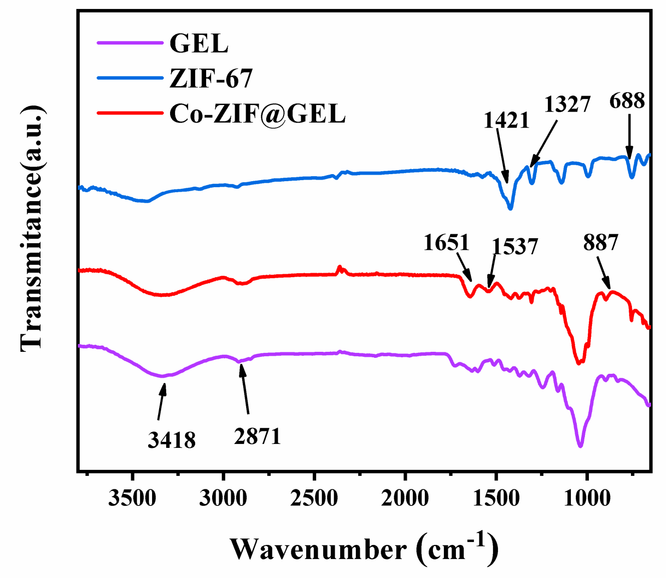

Figure S1. FTIR spectra of ZIF-67, Co-ZIF@GEL and cellulose aerogel.

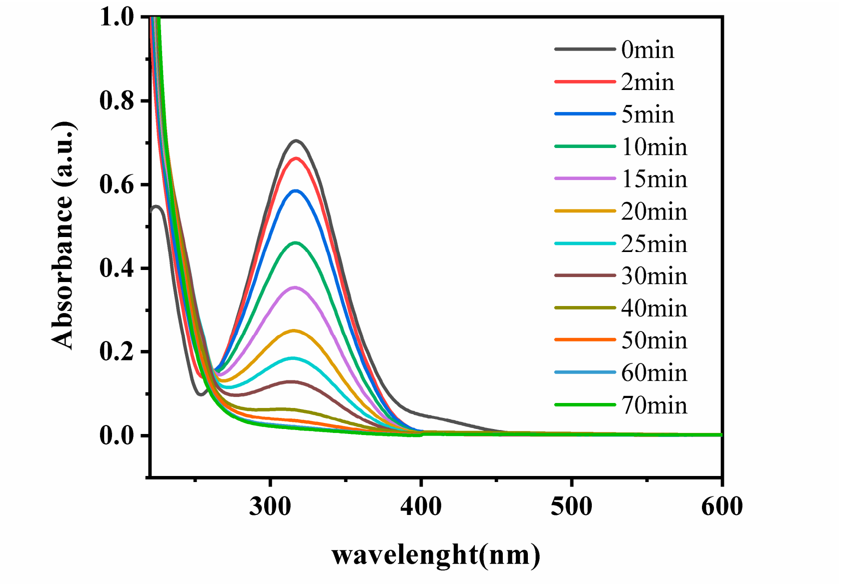

Figure S2. Absorption spectra for degradation of PNP in ZIF@GEL/PMS system. (condition: Co-ZIF@GEL= 100 mg/L, PMS=1 mM, PNP=10 mg/L, T=25°C;).
